# Compartmentalization of the Edinburgh Human Metabolic Network

**DOI:** 10.1186/1471-2105-11-393

**Published:** 2010-07-22

**Authors:** Tong Hao, Hong-Wu Ma, Xue-Ming Zhao, Igor Goryanin

**Affiliations:** 1 Department of Biochemical Engineering, School of Chemical Engineering & Technology, Tianjin University, Tianjin 300072, China; 2 Computational Systems Biology, School of Informatics, the University of Edinburgh, Edinburgh EH8 9AB, UK; 3 Edinburgh-Tianjin Joint Research Centre for Systems Biology and Synthetic Biology, Tianjin University, Tianjin 300072, China; 4 Key Laboratory of Systems Bioengineering, Ministry of Education, Tianjin University, Tianjin 300072, China; 5 Okinawa Institute of Science and Technology 1919-1, Onna, Onna-son, Okinawa 904-0412, Japan

## Abstract

**Background:**

Direct in vivo investigation of human metabolism is complicated by the distinct metabolic functions of various sub-cellular organelles. Diverse micro-environments in different organelles may lead to distinct functions of the same protein and the use of different enzymes for the same metabolic reaction. To better understand the complexity in the human metabolism, a compartmentalized human metabolic network with integrated sub-cellular location information is required.

**Results:**

We extended the previously reconstructed Edinburgh Human Metabolic Network (EHMN) [Ma, et al. Molecular Systems Biology, 3:135, 2007] by integrating the sub-cellular location information for the reactions, adding transport reactions and refining the protein-reaction relationships based on the location information. Firstly, protein location information was obtained from Gene Ontology and complemented by a Swiss-Prot location keywords search. Then all the reactions in EHMN were assigned to a location based on the protein-reaction relationships to get a preliminary compartmentalized network. We investigated the localized sub-networks in each pathway to identify gaps and isolated reactions by connectivity analysis and refined the location information based on information from literature. As a result, location information for hundreds of reactions was revised and hundreds of incorrect protein-reaction relationships were corrected. Over 1400 transport reactions were added to link the location specific metabolic network. To validate the network, we have done pathway analysis to examine the capability of the network to synthesize or degrade certain key metabolites. Compared with a previously published human metabolic network (Human Recon 1), our network contains over 1000 more reactions assigned to clear cellular compartments.

**Conclusions:**

By combining protein location information, network connectivity analysis and manual literature search, we have reconstructed a more complete compartmentalized human metabolic network. The whole network is available at http://www.ehmn.bioinformatics.ed.ac.uk and free for academic use.

## Background

Direct in vivo investigation of human metabolism is complicated by the distinct metabolic functions of different sub-cellular locations. For example, lysosomes are organelles containing digestive enzymes that break down polymeric macromolecules into their smaller building blocks. The lysosome membrane enables an acidic internal environment (pH less than 5 rather than around 7 in the cytosol) to maximize the enzyme activities [1]. Due to localization of metabolic enzymes, many metabolic processes involve coordinated interactions between different organelles, and one metabolic step may be dependent upon the successful completion of the previous step. For example, the decomposition of very long chain fatty acids (VLCFAs) is a process shared by peroxisomes and mitochondria. Likewise, the final steps in the synthesis of plasmologens occur in the endoplasmic reticulum, but the process depends on precursors which are synthesized in peroxisomes [2]. At the regulation level, the efficacy of many cellular processes is dependent on proper regulation of proteins trafficking to and from their site(s) of action. The endoplasmic reticulum (ER) and Golgi apparatus (GA) are known as the main organelles for protein targeting or protein sorting which transport proteins to the appropriate locations inside a cell or outside of it [3]. Therefore it is quite usual that an enzyme synthesized in ER may be active only in another sub-cellular location. In addition, diverse micro-environments in different organelles may lead to distinct functions of the same enzyme. For example, acid ceramidase (EC 3.5.1.23; AC) is the lipid hydrolase responsible for the degradation of ceramide into sphingosine and free fatty acids within lysosomes. However, at higher pH in the cytosol AC can also synthesize ceramide from sphingosine and free fatty acids [4]. Reduced lysosomal AC activity causes Farber disease, which is a member of a group of diseases called lysosomal storage diseases (LSDs) which results from defects in lysosomal enzyme function [5]. Therefore, determining the location of enzymes and reactions is important for the investigation of the mechanism of a metabolic process and its related diseases. Currently there are two high quality literature based human metabolic networks available, the Edinburgh Human Metabolic Network (EHMN) reconstructed by our group [6] and the Human Recon 1 reconstructed by Palsson's group [7]. Human Recon 1 contains eight sub-cellular locations while EHMN did not include location information [6]. Localization of reactions in Human Recon 1 was determined from "protein localization data, sequence targeting signals, and indirect physiological evidence". If these data were unavailable, reactions were assigned to cytoplasm (cytosol in the data downloaded from BiGG) [7]. As mentioned in our previous paper, EHMN is a more complete network with 1028 more reactions and 1202 more metabolites [6]. Therefore, it is valuable to EHMN to include the information on sub-cellular location distribution of proteins and reactions. In this work, we chose 8 locations for enzyme proteins mainly based on information from GO (Gene Ontology) [8]. The localization of reactions was initially determined by protein locations and then the gaps and isolated reactions in the preliminary compartmentalized network were examined and revised based on information from literature. By integrating the protein location information and the reaction (metabolite) location information, we also corrected hundreds of wrong protein-reaction relationships in EHMN. Furthermore, over 1400 transport reactions were added in order to obtain a connected compartmentalized network. The metabolic pathways for the synthesis and degradation of dozens of metabolites were investigated to validate the reconstructed network.

## Results and discussion

### 1. Localization based on protein location

#### 1.1 Protein location information

**Figure 1:**
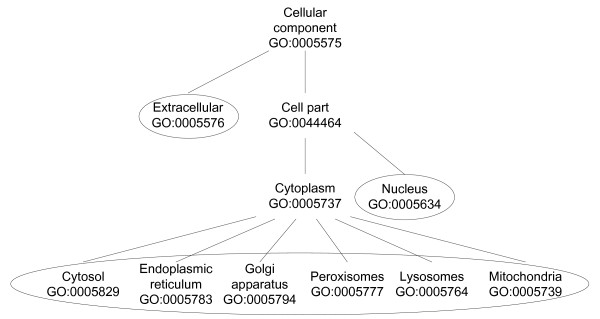
**The Gene Ontology terms used for protein locations**. The eight selected top locations are circled. Proteins are matched to GO terms and then backtracked to the eight selected locations through the hierarchical structure. "Cytosol" here includes the proteins on plasma membrane.

There are 15904 human proteins associated with one or more GO (Gene Ontology) terms in the "cellular component" part. They were classified into the eight selected high level locations including: cytosol, nucleus, Endoplasmic Reticulum (ER), Golgi apparatus (GA), peroxisomes, lysosomes, mitochondria and extracellular (See Methods and Figure 1). Proteins associated with "cell part" or some other locations not within the above eight locations were assigned to "uncertain". From Swiss-Prot keywords we obtained location information for 4908 human proteins and among them 154 proteins have no clear location information from GO. In the case that a protein has different locations in the two resources, we choose to assign the protein to all the locations. Altogether we obtained location information for 16058 human proteins and 2026 of them are in EHMN (summarized in Table 1). The high number of proteins allocated to "uncertain" reflects the fact that our knowledge on protein location distribution is still very limited.

**Table 1:** Protein distribution in different locations

GO	location	Number of proteins	Proteins in EHMN
GO:0005576	Extracellular	1969	145
GO:0044464	uncertain	12057	1606
GO:0005634	Nucleus	4668	391
GO:0005829	Cytosol	3782	380
GO:0005783	Endoplasmic reticulum	848	236
GO:0005794	Golgi apparatus	671	173
GO:0005777	Peroxisomes	99	51
GO:0005764	Lysosomes	178	41
GO:0005739	Mitochondria	911	324

#### 1.2 Sub-cellular localization of reactions

The reactions in EHMN were assigned to different locations based on the protein-reaction relationships and the obtained protein location information. There are also 448 non-enzyme catalyzed (or enzyme unknown) reactions and 143 reactions with unknown protein location information. They were assigned to "uncertain" location by default. In this way all the reactions in EHMN were assigned to the 9 locations and their distribution is shown in Table 2.

**Table 2:** The distribution of reactions in different locations

location	Number of reactions				
	
	original	after revision of type c reaction	after gap filling	after IRLR revision	after literature based revision
E	224	224	281	262	234
U	2481	2479	2163	2165	1929
N	218	218	265	252	226
C	650	650	775	726	892
ER	627	627	706	681	649
GA	228	228	255	248	241
X	378	360	386	376	291
L	123	123	134	116	108
M	793	779	877	866	740

**Figure 2:**
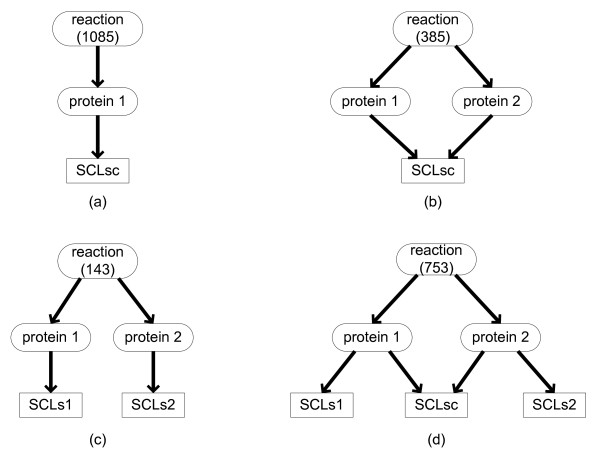
**The different types of protein-reaction-location relationships**. There are four types of protein-reaction-location relationships: (a) one reaction is related to one protein; (b) one reaction is related to two or more proteins which are all in the same locations; (c) one reaction is related to two or more proteins which are in completely different locations; (d) one reaction is related to two or more proteins which have common locations, but some of them also have their unique location annotation. SCLsc stands for the common locations. The number of reactions in each type is shown in the parentheses.

It should be noted that one reaction may be catalyzed by different enzymatic proteins and these proteins can be in different locations. For example, R00342 ((S)-Malate + NAD^+ ^= Oxaloacetate + NADH + H^+^) can be catalyzed by two proteins: MDH2 and MDH1, which function in cytosol and mitochondria respectively. Figure 2 shows a summary of 4 different types of protein-reaction-location relationships: One reaction may be catalyzed by one (type a) or multiple proteins. When a reaction is catalyzed by multiple proteins, these proteins can be active in the same (type b) or in different locations. For example, R00256 (L-Glutamine + H_2_O = L-Glutamate + NH_3_) is catalyzed by two proteins: GLS1 and GLS2. They both function in mitochondria but in different organs (kidney and liver) [9,10]. R00342 mentioned above belongs to type c where two proteins are in totally different locations. There are also cases in which some proteins for one reaction may have common locations but some of them may also have their unique locations (type d). For example, R05987 (UDP-N-acetyl-D-glucosamine + (GlcNAc)4 (Man)3 (Asn)1 = UDP + (GlcNAc)5 (Man)3 (Asn)1) is catalyzed by proteins MGAT4A, MGAT4B and MGAT4C. They all exist in GA but MGAT4A can also be secreted to extracellular space. However, the expression locations of these enzymes in tissues are somehow different although they have the common sub-cellular locations in cells. MGAT4A and MGAT4C are expressed in almost totally different organs. Whereas MGAT4B is widely expressed in many human tissues and highly over-expressed in pancreatic cancer [11].

We paid special attention to type c in the four types. Proteins catalyzing the same reaction in different locations are called "complementary proteins" in the study. We identified 16 such complementary protein groups (See additional file 1: Complementary groups and deleted protein-reaction relationships in revision of type c reaction). The two most common cases are: (a) the proteins are in the cytosol and mitochondria respectively (as for R00342 mentioned above); (b) the proteins are in peroxisomes and mitochondria respectively. Most of the protein groups in case b are involved in lipid metabolism and often catalyze a large class of reactions. As reported in literature, peroxisomes are the main places for the oxidation of VLCFAs [2]. Therefore the protein location information may be used to identify the correct reactions catalyzed and thus improve the annotation of the protein-reaction relationships in EHMN. For example, RE3139 (CoA + 3-oxo-all-cis-6,9,12,15,18,21-tetracosahexaenoyl-CoA = acetyl-CoA + docosa-4,7,10,13,16,19-all-cis-hexaenoyl-CoA) is a step in beta-oxidation of Omega-3 fatty acid, which is a family of long chain and very long chain unsaturated fatty acids and their oxidation was known to take place in peroxisomes [12]. RE3139 is associated with four proteins (ACAA1, ACAA2, HADHB and HADHA) in EHMN. However, only ACAA1 is in peroxisomes and the other three are mitochondria proteins. Therefore, the relationships between RE3139 and the three mitochondrial proteins in EHMN are wrong and should be corrected. Using this method, we have removed 43 incorrect protein-reaction relationships (See additional file 1: Complementary groups and deleted protein-reaction relationships in revision of type c reaction).

### 2. Location revision based on pathway connectivity analysis

According to the location of the reactions, we obtained the reaction location distribution for all the pathways in EHMN (See additional file 2: The reaction location distribution in the pathways in EHMN). We found that there are many poorly-studied pathways in which the number of reactions in any one specific location (excluding "uncertain") is less than one quarter of the total number of reactions in the pathway. This rather patchy feature of the localized network indicates that the location information from GO and SwissProt is still quite limited and more information is needed to improve the network for functional analysis. As the first step, we tried to improve the network connectivity by identifying the gaps or isolated reactions in the localized network by network structure analysis.

**Figure 3:**
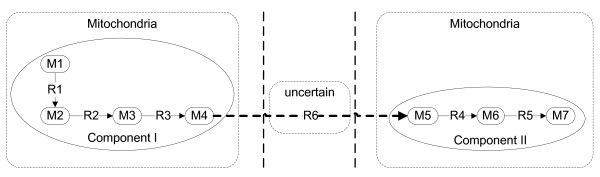
**An example of Gap filling based on pathway connectivity analysis**. The two connected components in Mitochondria can be linked by reaction R6 which is not in Mitochondria. Then R6 is a gap reaction and the gap could be filled by assign R6 to Mitochondria.

From additional file 2 we can also see that there are many cases in which only one isolated reaction in a pathway exists in one location. Even when there are several reactions in a pathway in the same location, they may be separated by a reaction in another location (defined as a gap) as shown in Figure 3. We need to address these issues to improve the quality of the compartmentalized network.

#### 2.1 Gap filling

As depicted in Figure 3, a gap is a reaction which is in a different location with its two neighbour reactions in a pathway. Details about the methods for gap identification can be found in the Methods section. Altogether we found 510 gaps from 360 reactions in the 69 pathways ("isolated" pathway was excluded). Interestingly, a major part of the gap reactions (316 of 360) are in "uncertain". We directly assigned these reactions to the corresponding location to fill the gaps. This gap filling method is similar to previous approaches to identify the gaps in a reconstructed metabolic network for searching the missing genes/enzymes in pathways [13-15]. Now we applied this connectivity-based gap filling method to the localized network, thus complementing the patchy location data obtained solely from protein location information.

For the 49 gaps whose gap reactions are not in "uncertain", we used the information from the literature to decide whether we should assign the gap reaction to the corresponding location or not. Only those with positive evidences in the literature to support the existence of a gap reaction in the missing location were filled. Due to the limited research on reaction location, we only found 8 gaps with positive evidence in the literature. For example, R03617 (Galactosylceramide + H_2_O = beta-D-Galactose + N-Acylsphingosine) was identified as a gap in extracellular whereas Dolcetta et al [16] reported that the corresponding protein GALC actually catalyzed the reaction in the extracellular space. For the other 7 reactions, we found some new proteins that can catalyze the reaction in the missing locations. For example, one of the gaps in the di-unsaturated fatty acid beta-oxidation pathway in peroxisomes can be filled by adding reaction RE1516 (FAD + linoleoyl-CoA = FADH_2 _+ trans,cis,cis-2,9,12-octadecatrienoyl-CoA). In EHMN, this reaction is catalyzed only by protein ACADL which is active in mitochondria. However, Bode and Couee [17] reported that two peroxisomal proteins: ACOX1 and ACOX3 have the same catalytic activity as ACADL. Therefore, RE1516 is catalyzed by ACOX1 and ACOX3 in peroxisomes. In this case, we updated EHMN by adding the protein-reaction relationships to the database. Altogether we added 14 protein-reaction relationships from literature and 469 gaps were filled, including 461 gaps from reactions with "uncertain" location and 8 gaps from reactions with other clear locations. The distribution of the reactions in each location after gap filling is shown in Table 2.

#### 2.2 Isolated reactions

**Figure 4:**
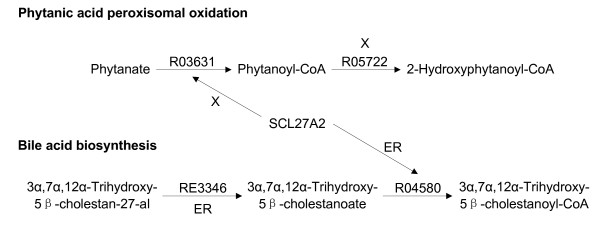
**Isolated reaction-location relationship revision**. Protein SLC27A2 is in both peroxisomes (X) and endoplasmic reticulum (ER) and it can catalyze two reactions: R03631 and R04580. R03631 is the only reaction in endoplasmic reticulum in the Phytanic acid peroxisomal oxidation pathway while there are other reactions connected with R04580 in endoplasmic reticulum in the Bile acid biosynthesis pathway. This strongly suggests that SLC27A2 may only catalyze R03631 in peroxisome but not in endoplasmic reticulum.

Gap filling may join some isolated reactions in a location together. However, even after gap filling, there are still some isolated reactions as not all reactions can be linked by a gap reaction. As for the gap definition, isolated reactions are also location-specific. One reaction may be isolated in two different locations. It can also be an isolated reaction in one location but not isolated in another location. More precisely it should be called an isolated reaction-location relationship (IRLR). Altogether we obtained 177 IRLRs by identifying the one-node connected components in the location-specific pathway graph and investigating them carefully in the literature. We found that some of them are wrong because of (1) incorrect reaction location annotation; (2) incorrect protein-reaction relationship in EHMN. In the first case, the existence of a protein in a location does not mean that the catalyzed reaction occurs in that location because the protein may be not active. For example, R03631 (ATP + Phytanate + CoA = AMP + Pyrophosphate + Phytanoyl-CoA) in the phytanic acid peroxisomal oxidation pathway is catalyzed by protein SLC27A2 which exists in both peroxisomes and endoplasmic reticulum [18]. However, many researches [19-21] reported that the oxidation of phytanic acid takes place in peroxisomes but not in endoplasmic reticulum. The activity of SLC27A2 in endoplasmic reticulum may be involved in the bile acid biosynthesis pathway to catalyze R04580 (ATP + 3alpha,7alpha,12alpha-Trihydroxy-5beta-cholestanoate + CoA = AMP + Pyrophosphate + 3alpha,7alpha,12alpha-Trihydroxy-5beta-cholestanoyl-CoA) (Figure 4) [22]. An example of the second case is R04586 (GM2 + H_2_O = GM3 + N-Acetyl-D-galactosamine), a GalNAc transformation reaction. In EHMN the corresponding protein for this reaction is MGEA5. However, the description of the function of MGEA5 in Swiss-Prot is "it Cleaves GlcNAc but not GalNAc from glycopeptides". Therefore the annotation for MGEA5 in EHMN was wrong and should be corrected. Altogether 7 protein-reaction relationships were deleted in this way.

For some isolated reactions, we found positive evidence in the literature. For example, the L-Pipecolic acid oxidation reaction R02204 (L-Pipecolate + Oxygen = 2,3,4,5-Tetrahydropyridine-2-carboxylate + H_2_O_2_) should occur in human peroxisomes. Its deficiency in this location will cause a disease belonging to a group of disorders called peroxisome biogenesis disorders (PBDs) [23]. Even though it is the only reaction in Lysine metabolism which occurs in peroxisomes, its location should not be changed, especially considering that oxygen is a reactant in this reaction. There are 19 such cases in our network. It is very likely that these reactions may be linked with other parts of the network through transport reactions.

We did not find any related literature for more than 80% of the IRLRs. In this case, we decided to keep an IRLR if the corresponding protein exists only in that location and it does not catalyze other reactions. As discussed above, if the protein exists in several locations, it may be inactive in the isolated location. Alternatively, if a protein catalyzes several reactions, it may imply that the protein may prefer to catalyze the other reaction(s) rather than the isolated one. For example, reaction R05989 (2 UDP-D-galactose + (GlcNAc)4 (LFuc)1 (Man)3 (Asn)1 = 2 UDP + (Gal)2 (GlcNAc)4 (LFuc)1 (Man)3 (Asn)1) in the N-Glycan biosynthesis pathway is an isolated reaction in the cytosol. Protein B4GALT1 is the protein related to this reaction in the cytosol but this protein also functions in extracellular and GA where it is connected with other reactions. In addition, B4GALT1 is responsible for the synthesis of complex-type N-linked oligosaccharides in many glycoproteins. Therefore, even if B4GALT1 has activity in the cytosol, its function may not be for reaction R05989 but for some other reactions. Accordingly, the location annotation of R05989 in the cytosol was deleted. However, further experimental studies would be needed to validate such predictions. In all the 177 isolated reaction-location relationships, 153 of them were revised, including 9 revised because of incorrect protein-reaction relationships and 144 without any related literature citations. The distribution of reactions in each location is shown in Table 2.

### 3. Literature-based revision

The objective of gap filling and IRLR revision is to make the compartmentalized network more amenable for further network analysis and functional analysis. However, even after such structure-based revision, there are still many pathways in which the number of reactions in any one specific location (excluding "uncertain") is less than one third of the total number of reactions in the pathway. To further improve the usability of the network, we manually examined the location distribution of all the pathways in EHMN, with a focus on pathway level, to check the literature for location information about a biological process including a series of reactions.

Although in total such information is still limited, we did find evidences in textbooks or in the literature for a number of pathways. In addition to the well known mitochondrial occurrence of the TCA cycle pathway, many studies are focused on the location of fatty acid beta-oxidation pathways. Three main criteria were used to decide the location of the beta-oxidation reaction. Firstly, it was reported that very long chain (greater than C-22) fatty acids underwent initial oxidation in peroxisomes which ceases at octanyl CoA and was then followed by mitochondrial oxidation [24]. Thus, the length of fatty acid chain can be considered as a rule to distinguish between beta-oxidation in mitochondria and in peroxisomes. Secondly, oxidation reactions coupled with ATP should occur in mitochondria while those coupled with H_2_O_2 _should be in peroxisomes [2]. The third criterion is the knowledge on the location of some specific metabolites. It has been reported that stearic acid and palmitic acid are oxidized firstly in peroxisomes to octanyl CoA and then oxidized in mitochondria. The unsaturated fatty acids (except very long chain acids) can be oxidized in both peroxisomes and mitochondria [2]. According to the above criteria, the locations of 103 reactions in the beta-oxidation pathways were revised. Due to the revised reaction location, the incorrect protein-reaction relationships were also corrected. For example, the location of R03856 (Lauroyl-CoA + NADP^+ ^= 2-trans-Dodecenoyl-CoA + NADPH + H^+^) was revised from both peroxisomes and mitochondria to peroxisomes only because it is a step in the degradation of palmitic acid in peroxisomes before ceasing at octanyl CoA. In EHMN, R03856 is linked with two proteins: MECR in mitochondria and PECR in peroxisomes. The MECR-R03856 relationship can therefore be deleted because the reaction only occurs in peroxisomes. In this way, a total of 129 protein-reaction relationships were deleted in the beta-oxidation pathways. We also revised the location information for 337 reactions in other pathways (e.g. Pentose phosphate pathway, Valine, leucine and isoleucine degradation pathway) based on evidences from textbooks or the literature and more than 120 incorrect protein-reaction relationships were deleted (See additional file 3: Result of literature-based revision). The reaction distribution in each location is shown in Table 2.

**Figure 5:**
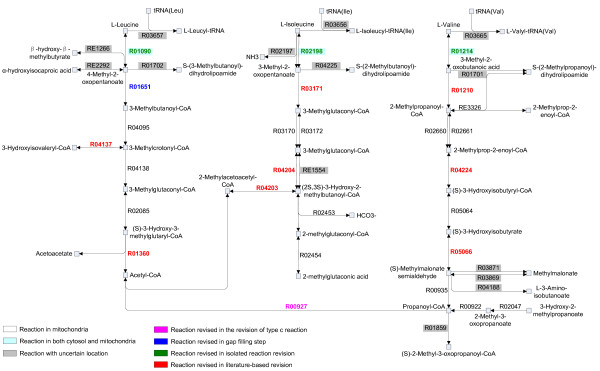
**An example pathway of compartmentalized human metabolic network: Valine, leucine and isoleucine degradation pathway**. The reactions in black and not highlighted are in mitochondria. Those highlighted in light blue are in both cytosol and mitochondria. The reactions with uncertain location (existing exclusively in "uncertain" or in "uncertain" besides mitochondria or cytosol) are highlighted in grey boxes. The reactions revised in the steps of revision of type c reaction, gap filling, IRLR revision and literature-based revision are in purple, dark blue, green and red, respectively.

The limited knowledge on sub-cellular location information from databases was remedied to some extent by the revisions described above. The Valine, leucine and isoleucine degradation pathway is shown in Figure 5 as an example to illustrate how the quality of the localized network was improved. Firstly, in the step of revision of type c reaction, the location of R00927 (Propanoyl-CoA + Acetyl-CoA = CoA + 2-Methylacetoacetyl-CoA) was revised from both mitochondria and peroxisomes to only mitochondria according to its chain length. Subsequently, in the gap filling step, we found that R01090 (L-Leucine + 2-Oxoglutarate = 4-Methyl-2-oxopentanoate + L-Glutamate) and R04095 (3-Methylbutanoyl-CoA + FAD = 3-Methylcrotonyl-CoA + FADH_2_) in this pathway were in two different components in mitochondria, respectively. These two reactions can actually be connected by R01651 (4-Methyl-2-oxopentanoate + CoA + NAD^+ ^= 3-Methylbutanoyl-CoA + CO_2 _+ NADH) whose original location is "uncertain". Therefore R01651 was classified as a gap reaction to fill the gap between R01090 and R04095. With this gap filled, the L-Leucine degradation pathway in mitochondria was completed. Similarly, we found that the three reversible transamination reactions catalyzed by branched-chain amino-acid aminotransferase (BCAT): R01090 (L-Leucine + 2-Oxoglutarate = 4-Methyl-2-oxopentanoate + L-Glutamate), R02198 (L-Isoleucine + 2-Oxoglutarate = 3-Methyl-2-oxopentanoate + L-Glutamate) and R01214 (L-Valine + 2-Oxoglutarate = 3-Methyl-2-oxobutanoic acid + L-Glutamate), were isolated reactions in the nucleus. Two BCATs exist in mammalian cells: BCATm in mitochondria and BCATc in the cytosol. BCATm functions in brain and kidney while BCATc is ubiquitous in most human tissues [25]. Therefore, this step can occur in both mitochondria and cytosol but not in the nucleus. Therefore we revised the locations of these three reactions to cytosol and mitochondria. In addition, Hutson et al reported that all the steps which commit the BCAA carbon skeleton to the degradation pathway take place in mitochondria [26,27]. Based on this information, eight reactions in the degradation pathway (R04203, R04204, R05066, R04137, R04224, R03171, R01210, R01360), which also exist in several other locations besides mitochondria (such as "uncertain", cytosol and peroxisomes), were relocated to be exclusively in mitochondria. From this example, we can see that the structure-based and literature-based revisions do greatly improve the location distribution of the pathways.

### 4. Transport reactions

It is important to include transport reactions between different compartments in a localized network in order to make the network connected. It is unreasonable to add a transport reaction involving the "uncertain" location. Therefore we assigned all the reactions with "uncertain" location to cytosol as that used in Recon 1 [28]. As Recon 1 already included transport reactions, we first took it as a reference to add the transport reactions to our network. There are 1190 transport reactions in Recon 1 and 857 of them are for metabolites that are also in EHMN. Among the 857 reactions, only 378 reactions that are based on biochemical or genetic experiments (with confidence score 3 in Recon 1) were directly added into EHMN. For the 310 reactions based on physiological data or biochemical/genetic evidence from a nonhuman mammalian cell (with confidence score 2 in Recon 1), a transport reaction was added only if the corresponding metabolite exists in both compartments involved in the transport reaction. The reactions with confidence score less than 2 in Recon 1 were ignored because those reactions were added merely based on *in sillico *simulation. Finally 546 transport reactions were added from Recon 1 and 429 of them are related with 212 transport proteins.

At the second step, we searched human transport proteins in TransportDB [29] and all the proteins with gene ontology annotation GO: 0005215 (transporter activity) in Uniprot. There are over 1000 transport proteins obtained from the two resources. However, a large number of the proteins are ion channel related proteins or proteins annotated based on sequence similarity but without clear substrate information. Excluding the 212 transport proteins already obtained from Recon 1, only a small proportion of the transport proteins were investigated to add corresponding transport reactions based on information from the databases and literature. Altogether 109 new transport reactions from 83 transport proteins were added to EHMN. Some of them are actually also in Recon 1 but with a low confidence score and without assigned transport protein.

After adding 655 reactions based on Recon 1 and transporter information from databases and literature, there are still many gaps between different compartments in the network mainly because of our limited knowledge on the function of transport proteins and the existence of non-protein required transport reactions. To make the network connected and thus amenable for pathway analysis, we calculated the "dead ends" in each location for each pathway (excluding the isolated pathway) and tried to link the dead ends in different locations by adding new transport reactions. A dead end was defined as a metabolite that is only produced or consumed in a location. If a dead end metabolite is also appeared in other locations then transport reactions are added for it. By default, cytosol was used as a bridge to connect other locations and transport reaction to/from cytosol were added for the dead end metabolites. Altogether 961 transport reactions were identified for the dead end metabolites. Among them, 193 reactions were already added in the first two steps (from Recon 1 and transport proteins). Therefore 768 new transport reactions were needed to make the network connected. It should be noted that the reactions added solely based on dead end analysis are of low quality and the existence are subjected to further experimental investigation. Among these reactions, 73 are between cytosol and nucleus which actually can be carried out by the nuclear pore complexes formed by the 49 nucleoporin proteins in human [30].

As a summary 1423 transport reactions were added to the network and 611 of them are associated with transport proteins. The whole network containing 4804 reactions is available at http://www.ehmn.bioinformatics.ed.ac.uk and free for academic use.

### 5. Comparison of EHMN with other human metabolic networks

Besides EHMN and Recon 1, human metabolic networks are also available from Reactome [31] and HumanCyc [32]. HumanCyc is a computationally reconstructed network including 1716 reactions (May 2010 version). However there is no location information for reactions included in HumanCyc. Therefore, we mainly compared the two compartmentalized human metabolic networks (Recon 1 and Reactome) with EHMN. Reactome contains both metabolic pathways and signal pathways. Only the 998 reactions in the 12 metabolic pathways were extracted (see notes of Table 3). Reactions in Recon 1 were obtained from the BiGG database. The numbers of reactions in different compartments of the three networks are shown in Table 3. It can be seen that comparing with EHMN and Recon 1, the human metabolic network from Reactome is small. Especially the reaction information in the compartments other than cytosol and mitochondria is rather patchy. Due to the different compound names used in the three networks, it is very difficult to check which parts of the network are common in all three networks. Actually by matching the compounds through common KEGG compound IDs or exact name we only found 409 common compounds between EHMN and Reactome and 821 between EHMN and Recon 1. To match reactions between two networks all the reactants involved in the reaction need to be matched. Therefore we got even smaller numbers of matching reactions (229 between EHMN and Reactome; 578 between EHMN and Recon 1). Many of the common reactions are in the central pathways, nucleotide metabolism and metabolic pathways for some amino acids. However, the small number of found common reactions makes it very difficult for a comprehensive comparison of the networks. Generally, EHMN has more metabolic reactions which are allocated to clear locations (3381 *vs *2147) than Recon 1. By comparing the pathway maps of Recon 1 (supplementary figures 7, 8, 9, 10, 11, 12, 13, 14 in ref [28]) and those from EHMN http://www.ehmn.bioinformatics.ed.ac.uk/pathway/, we can find that many reactions in the lipids metabolism pathways (ex. linoleate degradation, omega-3 and omega-6 fatty acid beta-oxidation and synthesis of some prostaglandins) in EHMN are missing in Recon 1. There are also some reactions which are in both networks but are assigned to different locations. For example, R01975 (3-Hydroxybutanoyl-CoA + NAD^+ ^= Acetoacetyl-CoA + NADH) is a step of Saturated fatty acids beta-oxidation pathway after octanyl CoA, which is localized to mitochondria in EHMN but not in peroxisomes according to literature [2]. In Recon 1, however, this reaction occurs in both peroxisomes and mitochondria. R01324 (Citrate = Isocitrate) was assigned to cytosol, GA, ER and mitochondria in EHMN whereas it is only in cytosol and mitochondria in Recon 1. The extra locations of GA and ER in EHMN are based on the new evidence that the corresponding enzyme IRP1 is also in the two locations [33].

**Table 3:** Sub-cellular location comparison of Human Recon 1, EHMN, and Reactome database

	Human Recon 1^a^	Reactome^b^	EHMN
Metabolic reactions	2147	809(998)^c^	3381 (4793)^d^
Extracellular	53	52	234
Nucleus	87	6	226
Cytosol	957	440(629)^c^	892 (2304)^d^
Endoplasmic reticulum	187	64	649
Golgi apparatus	253	1	241
Peroxisomes	92	43	291
Lysosomes	190	0	108
Mitochondria	328	203	740
Non-repeat metabolic reactions^e^	1836	998	2806
Transport reactions	1190 (1596)^f^	218	1423

^a ^the numbers is based on the data downloaded from BIGG database and may be different from those in their published paper. ^b ^the metabolic reactions from Reactome include metabolism of amino acids and derivatives, carbohydrates, nitric oxide, nucleotides, polyamines, porphyrins, vitamins and cofactors, pyruvate and citric acid cycle, lipids and lipoproteins, energy, biological oxidations and electron transport chain. The transport reactions include membrane trafficking and transmembrane transport of small molecules pathways. ^c ^the number in the parentheses includes the reactions which were assigned to "cytosol" from unclear location or locations other than the chosen ones. ^d ^the number in the parentheses includes the reaction which are assigned to "cytosol" from "uncertain". ^e ^a reaction occurs in different locations was counted only once. ^f ^the number in the parentheses includes the exchange reactions.

### 6. *Function analysis *of the compartmentalized EHMN

**Figure 6:**
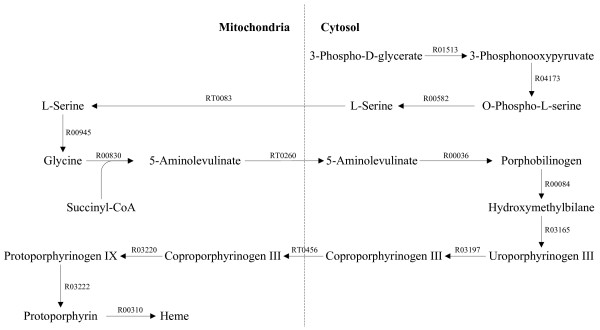
**The Heme synthesis pathway across different locations**.

To validate the reconstructed network, we have systematically analyzed the pathways for the synthesis and degradation of certain key metabolites using a newly developed pathway analysis tool (available on http://www.ehmn.bioinformatics.ed.ac.uk/path/). Around 70 metabolic conversion processes were examined including the synthesis and degradation of amino acids, ribonucleotides, deoxyribonucleotides, fatty acids, sterols, glycans, prostaglandins and heme (additional file 4: *In silico *metabolic capability analysis of the compartmentalized EHMN). All the pathways can be visually inspected through the hyperlinks in the additional file. Actually, pathways between any two metabolites can be checked through the pathway analysis tool and this tool has been used in manual examination of our network. In most cases, the synthesis/degradation pathways found are in consistent with those reported in literature or textbooks. Taking the synthesis of heme as an example (Figure 6), L-Serine is synthesized in the cytosol and then transported into mitochondria for the production of 5-Aminolevulinate. Then 5-Aminolevulinate is transported back into the cytosol for the further synthesis of Coproporphyrinogen III. The final steps from Coproporphyrinogen III to heme take place in mitochondria again [34,35].

## Conclusions

A compartmentalized human metabolic network has been reconstructed by adding the sub-cellular location information to enzymes and reactions in EHMN. Network structure analysis and manual curation of literature were used to improve the quality of the localized network which was mainly based on protein location information. Transport reactions were added to obtain a connected sub-cellular location specific metabolic network. The refined network was validated by *in silico *investigation of the metabolic functions for the production/degradation of certain important metabolites.

## Methods

### Localization of human proteins

The protein location information in this work is extracted mainly from Gene Ontology (GO). Files containing gene-GO association in human and the hierarchically organized GO terms ("OBO" file) can be easily downloaded from the GO website. This file is maintained by the GOA group at EBI which aims to provide high-quality GO annotations to proteins in the UniProtKB. GO database provides a controlled vocabulary of hierarchically organized terms to describe the location in the "cellular component" category. There are over one thousand GO terms in the "cellular component" category. We chose eight top locations to compartmentalize the EHMN based on the cellular structure. The selected locations and their hierarchical structure are shown in Figure 1. The organelles considered as individual compartments are nucleus, Endoplasmic Reticulum (ER), Golgi apparatus (GA), peroxisomes, lysosomes and mitochondria. Other organelles such as microsome, cytoskeleton and small vesicles were not considered because their functions are often not related with cellular metabolism. The term "plasma membrane" in GO was merged with "cytosol" in this work, so all the proteins which are annotated in "plasma membrane" in GO were classified to "cytosol" because they usually catalyze reactions occurring in the cytosol. From the gene-GO association file, many proteins are actually associated with a subordinate term such as mitochondrial membrane (GO: 0031966). In these cases, we traced the GO tree back to a higher-level term within the eight chosen locations (mitochondria in this case). The proteins with uncertain locations or other locations not included in the eight specific locations were classified to an "uncertain" location. In this way, all the human proteins coming from GO were classified into the chosen locations.

To obtain more protein location information and cross-validate information from different sources, we also used the Swiss-Prot keywords to extract all the human proteins related with the keywords. There are more than eighty locations listed as keywords in the "cellular component" category in Swiss-Prot keywords, in which six locations, including nucleus, ER, GA, peroxisomes, lysosomes and mitochondria, are same with the GO terms used in our study. We extracted all the human proteins related with these location keywords to get the protein-location relationships.

### Gap filling

We developed a method to automatically identify gaps in the network. For each pathway (excluding isolated pathway), we extracted a subset of reactions which are in a specific location and then converted it into a graph. The group of reactions in a location which can be connected were classified into one component, so the reactions in a location may be divided into several separated components. It should be noted that only the main compounds in a reaction were used in linking reactions and the currency metabolites were ignored [36]. Then we calculated the connected components in the reaction graph [37] and obtained a list of main metabolites in each component. In the next step, we checked every reaction which is not in this location to see if it contains main metabolites in two different components. If so, this reaction will be identified as a gap for that location. This process was repeated for each location (excluding "uncertain") and one reaction may be identified as gaps in different locations. A gap is a missing reaction-location relationship rather than a reaction.

## Abbreviations

GO: gene ontology; IRLR: isolated reaction-location relationship; E: Extracellular; U: uncertain; N: Nucleus; C: Cytosol; ER: Endoplasmic reticulum; GA: Golgi apparatus; X: Peroxisomes; L: Lysosomes; M: Mitochondria; VLCFA: Very long chain fatty acid.

## Authors' contributions

TH designed and performed the experiments, and wrote the manuscript. HM provided the overall project guidance, critical review and gave advices on the experiment and validation. XZ and IG gave advices on the validation. All authors read and approved the manuscript.

## Supplementary Material

Additional file 1 **Complementary groups and deleted protein-reaction relationships in revision of type c reaction**. Sheet 1 in this file contains the 16 complementary groups. Sheet 2 contains the 43 deleted protein-reaction relationships in the revision of type c reaction. Click here for file

Additional file 2 **The reaction location distribution in the pathways in EHMN**. Rows in the matrix stand for the pathways and columns stand for the locations. A yellow square indicates that there is only one reaction in a pathway in the corresponding location, while a blue square means that all the reactions in a pathway are in the corresponding location. The gray scale reflects the percentage of reactions in a pathway in the location. Abbreviations: E, Extracellular; N, Nucleus; C, Cytosol; ER, Endoplasmic reticulum; GA, Golgi apparatus; X, Peroxisomes; L, Lysosomes; M, Mitochondria. Click here for file

Additional file 3 **Result of literature-based revision.** Sheet 1 includes basic information of location revised reaction (ID, reversibility, equation, pathway), the reaction location before revision and that after revision. The reaction in beta-oxidation is marked in column 8 and the corresponding textbooks and literature are listed in column 9. Sheet 2 contains the deleted protein-reaction relationships. The protein-reaction relationships deleted in beta-oxidation revision are marked in column 5. Click here for file

Additional file 4 ***In silico *metabolic capability analysis of the compartmentalized EHMN**. The *in silico *inspection results of 68 metabolic processes are listed. The hyperlinks for visually checking the pathways using our web-based pathway analysis tool are included. Click here for file
