# Supplementary material for: Compartmentalization of the Edinburgh Human Metabolic Network
Source: BMC Bioinformatics. 2010 Jul 22;11:393. doi: 10.1186/1471-2105-11-393 (PMC2918583; doi:10.1186/1471-2105-11-393)
Supplement: Additional file 2 — The reaction location distribution in the pathways in EHMN. Rows in the matrix stand for the pathways and columns stand for the locations. A yellow square indicates that there is only one reaction in a pathway in the corresponding location, while a blue square means that all the reactions in a pathway are in the corresponding location. The gray scale reflects the percentage of reactions in a pathway in the location. Abbreviations: E, Extracellular; N, Nucleus; C, Cytosol; ER, Endoplasmic reticulum; GA, Golgi apparatus; X, Peroxisomes; L, Lysosomes; M, Mitochondria. [file 1471-2105-11-393-S2.PDF]

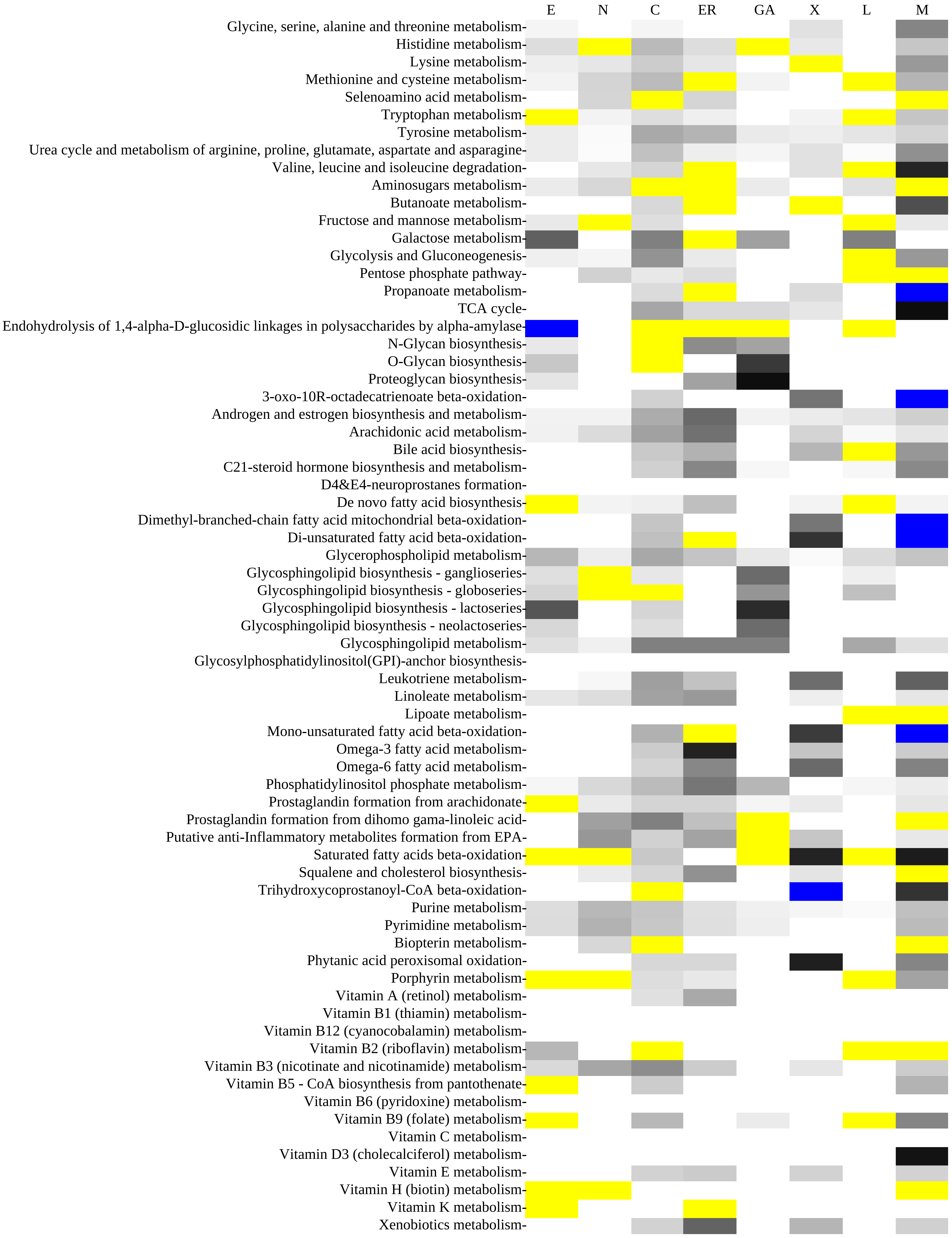

E
N
C
ER
GA
X
L
M
Glycine, serine, alanine and threonine metabolism
Histidine metabolism
Lysine metabolism
Methionine and cysteine metabolism
Selenoamino acid metabolism
Tryptophan metabolism
Tyrosine metabolism
Urea cycle and metabolism of arginine, proline, glutamate, aspartate and asparagine
Valine, leucine and isoleucine degradation
Aminosugars metabolism
Butanoate metabolism
Fructose and mannose metabolism
Galactose metabolism
Glycolysis and Gluconeogenesis
Pentose phosphate pathway
Propanoate metabolism
TCA cycle
Endohydrolysis of 1,4-alpha-D-glucosidic linkages in polysaccharides by alpha-amylase
N-Glycan biosynthesis
O-Glycan biosynthesis
Proteoglycan biosynthesis
3-oxo-10R-octadecatrienoate beta-oxidation
Androgen and estrogen biosynthesis and metabolism
Arachidonic acid metabolism
Bile acid biosynthesis
C21-steroid hormone biosynthesis and metabolism
D4&E4-neuroprostanes formation
De novo fatty acid biosynthesis
Dimethyl-branched-chain fatty acid mitochondrial beta-oxidation
Di-unsaturated fatty acid beta-oxidation
Glycerophospholipid metabolism
Glycosphingolipid biosynthesis - ganglioseries
Glycosphingolipid biosynthesis - globoseries
Glycosphingolipid biosynthesis - lactoseries
Glycosphingolipid biosynthesis - neolactoseries
Glycosphingolipid metabolism
Glycosylphosphatidylinositol(GPI)-anchor biosynthesis
Leukotriene metabolism
Linoleate metabolism
Lipoate metabolism
Mono-unsaturated fatty acid beta-oxidation
Omega-3 fatty acid metabolism
Omega-6 fatty acid metabolism
Phosphatidylinositol phosphate metabolism
Prostaglandin formation from arachidonate
Prostaglandin formation from dihomo gama-linoleic acid
Putative anti-Inflammatory metabolites formation from EPA
Saturated fatty acids beta-oxidation
Squalene and cholesterol biosynthesis
Trihydroxycoprostanoyl-CoA beta-oxidation
Purine metabolism
Pyrimidine metabolism
Biopterin metabolism
Phytanic acid peroxisomal oxidation
Porphyrin metabolism
Vitamin A (retinol) metabolism
Vitamin B1 (thiamin) metabolism
Vitamin B12 (cyanocobalamin) metabolism
Vitamin B2 (riboflavin) metabolism
Vitamin B3 (nicotinate and nicotinamide) metabolism
Vitamin B5 - CoA biosynthesis from pantothenate
Vitamin B6 (pyridoxine) metabolism
Vitamin B9 (folate) metabolism
Vitamin C metabolism
Vitamin D3 (cholecalciferol) metabolism
Vitamin E metabolism
Vitamin H (biotin) metabolism
Vitamin K metabolism
Xenobiotics metabolism
